# Osteoarthritis: Insights Offered by the Study of Bone Mass Genetics

**DOI:** 10.1007/s11914-021-00655-1

**Published:** 2021-02-04

**Authors:** A. Hartley, C. L. Gregson, L. Paternoster, J. H. Tobias

**Affiliations:** 1 Musculoskeletal Research Unit, Translational Health Sciences, Bristol Medical School, University of Bristol, Bristol, UK; 2 MRC Integrated Epidemiology Unit, Bristol Medical School, University of Bristol, Bristol, UK

**Keywords:** Osteoarthritis, Bone mineral density, High bone mass, Genetics

## Abstract

**Purpose of Review:**

This paper reviews how bone genetics has contributed to our understanding of the pathogenesis of osteoarthritis. As well as identifying specific genetic mechanisms involved in osteoporosis which also contribute to osteoarthritis, we review whether bone mineral density (BMD) plays a causal role in OA development.

**Recent Findings:**

We examined whether those genetically predisposed to elevated BMD are at increased risk of developing OA, using our high bone mass (HBM) cohort. HBM individuals were found to have a greater prevalence of OA compared with family controls and greater development of radiographic features of OA over 8 years, with predominantly osteophytic OA. Initial Mendelian randomisation analysis provided additional support for a causal effect of increased BMD on increased OA risk. In contrast, more recent investigation estimates this relationship to be bi-directional. However, both these findings could be explained instead by shared biological pathways.

**Summary:**

Pathways which contribute to BMD appear to play an important role in OA development, likely reflecting shared common mechanisms as opposed to a causal effect of raised BMD on OA. Studies in HBM individuals suggest this reflects an important role of mechanisms involved in bone formation in OA development; however further work is required to establish whether the same applies to more common forms of OA within the general population.

## Introduction

Osteoarthritis (OA) and osteoporosis are the two most prevalent musculoskeletal disorders globally, responsible for high levels of disability and healthcare utilisation, for example, through joint replacements and fractures, respectively [1]. OA is recognised as a disease of the whole joint and is considered a disorder of the bone-cartilage unit [2]. Cross-sectional studies consistently find that osteoporosis is inversely related to osteoarthritis (OA), as evidenced by positive associations between bone mineral density (BMD) and prevalent radiographic hip [3–7] and knee [8, 9] OA. Conversely, an inverse relationship has been observed between BMD and progression of knee OA [10, 11]. This may reflect a role of increased bone turnover, which is usually inversely related to BMD and is also associated with OA progression. For example, in the UK Chingford study, Bettica et al. observed that levels of the bone resorption marker, urinary NTX, were higher in progressive compared to that in non-progressive cases [12]. The suggestion that bisphosphonates, which suppress bone turnover and increase BMD, ameliorate some aspects of OA symptoms supports the suggestion that increased bone turnover contributes to OA pathogenesis [13]. This apparent paradox, whereby increased BMD contributes both positively and negatively to OA pathogenesis, may reflect distinct mechanisms according to the stage of OA development. For example, increased subchondral bone remodelling, involving the subchondral plate and subjacent cancellous bone, may contribute to OA initiation [14]. In contrast, subchondral sclerosis, a feature of relatively advanced OA, results from subsequent thickening and increased density of subchondral bone immediately adjacent to the joint and may contribute to further cartilage loss due to a loss of compliance leading to increased strains on the nearby cartilage [15].

In light of these well-established, albeit complex, relationships between BMD and OA, the study of bone genetics may offer important insights into the pathogenesis of OA. As well as identifying possible shared molecular mechanisms, genetic understanding may help in unravelling causal pathways. For example, studying whether those genetically predisposed to raised BMD are at increased risk of OA provides a stronger basis for making causal inferences compared with observational studies, inherently prone to residual confounding.

## High Bone Mass and Osteoarthritis

### OA Risk in Individuals with HBM

Over the last decade, we have studied our unique population of individuals with HBM to examine a possible causal role of raised BMD in the development of OA; these HBM individuals have a generalised increased BMD, more than 3 standard deviations above average for their age, which is clinically unexplained [16]. Our studies have been based on the premise that HBM individuals are genetically predisposed to their raised BMD, which manifests in young adulthood. Hence, raised BMD is expected to precede any subsequent development of OA, in keeping with a causal relationship. We initially found that individuals with HBM are at increased risk of developing OA, with a 4.5 times higher odds of partial or total hip replacement compared with that in family controls [17]. HBM individuals also had a greater prevalence of joint replacement at any site compared with general population data from the Health Survey for England [17]. We subsequently examined the risk of prevalent OA in the HBM population based on radiographs obtained from these individuals, using age- and sex-matched general population controls from Chingford and Hertfordshire cohort studies. An increased risk of hip and knee radiographic OA was observed [18, 19]. Body mass index (BMI) is an important confounder when examining BMD and OA relationships, since BMI is positively associated with both. This may well contribute to associations between HBM and OA, given our finding that BMI is significantly increased in HBM individuals [20]. Adjustment for BMI made little difference to the results for hip OA but attenuated the OR for the effect of HBM on knee OA from 2.38 to 1.62, suggesting the increased fat mass of HBM individuals partially contributes to the relationship between HBM and knee OA [20].

When analysing the individual radiographic sub-phenotypes of OA (osteophytes, joint space narrowing [JSN], subchondral sclerosis), an increased odds of osteophytes in HBM individuals was observed at both hip and knee joints; plus an increased odds of subchondral sclerosis was observed at the hip. However, there was no difference in odds of JSN between individuals with and without HBM at either joint [18, 19]. The same pattern of increased odds of osteophytes but not JSN in individuals with HBM compared to unaffected relatives was also observed in the (non-weight-bearing) distal interphalangeal (DIP) and carpometacarpal (CMC) joints of the hand [21]. Recently, we were able to examine the relationship between HBM and subsequent OA incidence/progression. We found that HBM is associated with an increase in knee osteophyte and JSN score over an average of 8 years, as well as knee pain and functional limitation [22]. Similar results have also been observed at the hip (A Hartley et al., unpublished data). Therefore, contrary to findings from conventional observational studies, it would seem that raised BMD contributes to OA pathogenesis across all phases of disease.

**Fig. 1:**
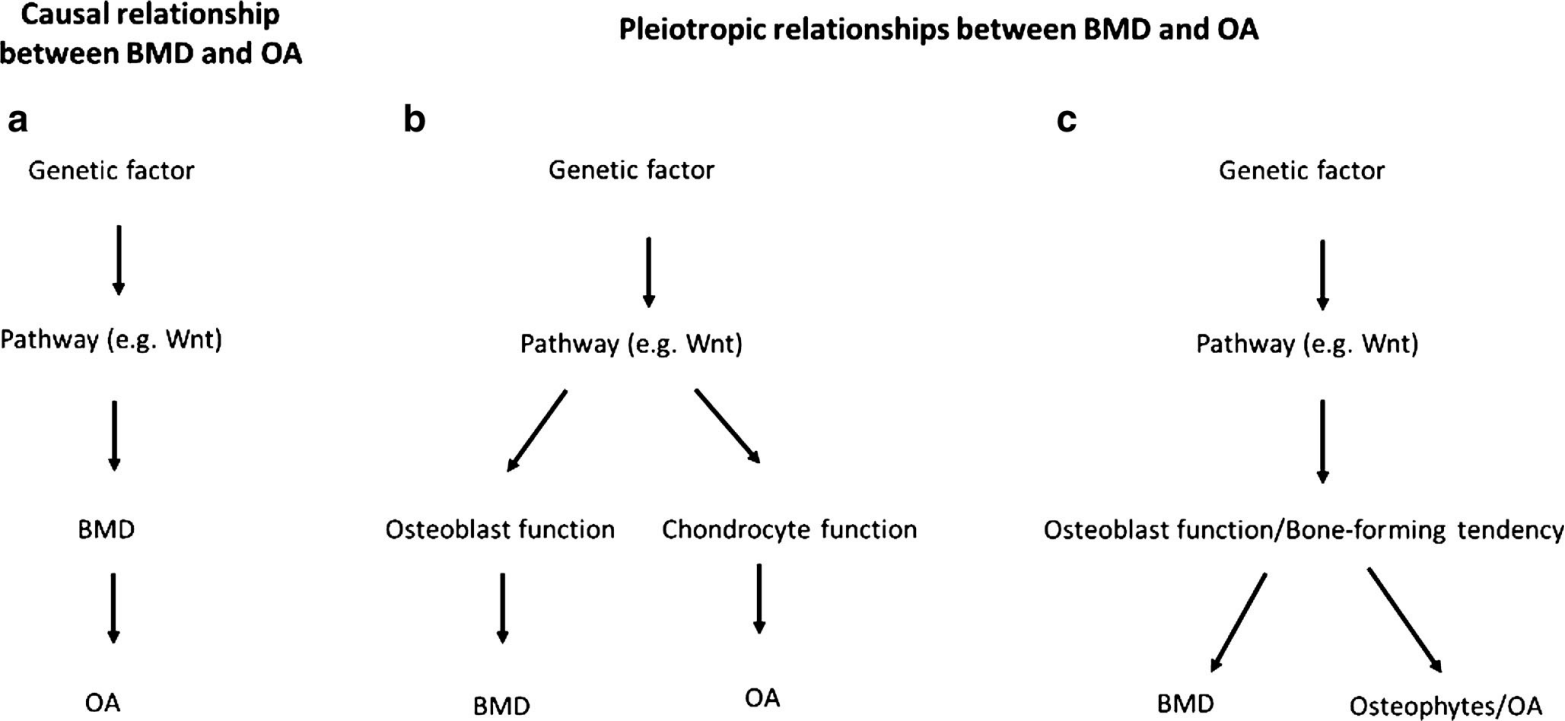
Potential mechanisms for the increased risk of OA which we observed in HBM individuals. (A) Causal: raised BMD in HBM increases the risk of OA as a consequence of a causal effect of BMD on OA—in this scenario, genetic variants from any pathway that influences BMD would also be associated with OA. (B) Pleiotropy 1: pathways are affected which influence both osteoblast function and chondrocyte function, leading to an increase in BMD and OA risk, respectively—in this scenario, we might expect only genetic variants from specific BMD-influencing pathways to be associated with OA. (C) Pleiotropy 2: HBM is associated with a ‘bone-forming’ tendency, which results in increased BMD and a higher risk of OA due to greater susceptibility to osteophyte formation—in this scenario, though BMD is not the trait that causally effects OA (a proximal intermediate trait is), it is less likely that there will be genetic variants associated with BMD that are not also associated with OA, making this scenario difficult to distinguish from scenario (A)

There are several potential mechanisms for the increased risk of OA which we observed in HBM individuals. If raised BMD is on the causal pathway for OA (Fig. 1a), this would be consistent with a role of increased subchondral density and stiffness as an initiating event in OA development, first proposed by Radin [23]. However, this explanation is now thought less likely following detailed study of temporo-spatial changes during OA development [14]. On the other hand, genetic factors related to BMD might lead to OA via a separate, pleiotropic pathway. This could be due to a genetic variant operating either through multiple pathways or through a particular pathway having direct effects on both phenotypes. For example, as discussed below, the Wnt signalling pathway regulates both osteoblasts and chondrocytes, leaving the possibility that the same genetic influences on BMD lead to OA via separate effects on the cartilage (Fig. 1b). Alternatively, to the extent that HBM reflects an underlying tendency towards increased osteoblast activity and bone formation, the same tendency might increase the risk of developing OA as a consequence of greater susceptibility to osteophyte formation (Fig. 1c). The possible application of Mendelian randomisation (MR) to distinguish these scenarios is discussed under ‘Future Work’.

### HBM and the ‘Bone-Forming’ Phenotype

There are several lines of evidence showing that individuals from the HBM cohort have a tendency towards excess bone formation. For example, an increased prevalence of pelvic radiographic enthesophytes (formation of extra bone at tendon or ligament insertions) was observed in these individuals [24]. In addition, mutations in *LRP5* are thought to lead to HBM through over-stimulation of bone formation [25, 26]. Similarly, the *SMAD9* mutation, which we recently identified in association with HBM, is predicted to cause over-stimulation of osteoblast differentiation [27]. Our HBM population also possesses an over-representation of BMD-related alleles, several of which are implicated in increased osteoblast activity, including the novel BMD-associated loci *NPR3* and *SPON1* [28]. Consistent with the suggestion of a phenotype resulting from excess bone formation, HBM cases show evidence of increased periosteal bone formation as evidenced by greater periosteal circumference as assessed by peripheral quantitative computer tomography (pQCT) [29].

As well as the suggestion that increased BMD in HBM individuals likely arises from increased bone formation, the same may apply to their OA phenotype, given the stronger association of HBM with osteophytes as opposed to JSN. OA is recognised to have several sub-phenotypes, and it may be that increased BMD in our HBM population is responsible for a predominantly osteophytic form of disease. That said, though osteophytes are a recognised feature of OA, their precise role in OA pathogenesis remains unclear. Though osteophytes are often observed in the context of cartilage loss and subsequent biomechanical changes, they may have an adaptive role in joint stabilisation rather than actively contributing to further deterioration. In addition, the extent to which osteophytes contribute to clinical features such as pain remains to be established. However, our recent finding that adjustment for knee osteophyte severity attenuated the relationship between HBM and knee pain scores provides some evidence that osteophytes contribute to pain in OA [22]. Moreover, HBM appears to be associated with true symptomatic OA, in light of their increased risk of joint replacement as discussed above [17].

## Shared Genetic Factors Between Bone and OA

The study of bone genetics has proved useful in identifying shared regulatory pathways which might contribute to the association between OA and BMD/osteoporosis. The bone and cartilage share several developmental and regulatory pathways, including the Wnt pathway and transforming growth factor beta (TGF-β) superfamily [2]. Genetic disorders affecting the Wnt pathway have been implicated in both OA and osteoporosis (see below); likewise loci in several genes linked to the TGF-β superfamily have been identified as being related to the risk of OA, including *GDF5*, *BMP2*, *TGFβ1*, *SMAD3* and *ASPN* [30–34]. Hence, common genetic influences on BMD and OA might be expected, as a consequence of genetic variation in these shared pathways. This question was examined specifically by Hackinger et al., who studied the genetic overlap between OA and BMD, based on genome-wide data from GEFOS (BMD) and arcOGEN (OA) [30]. Significant genetic correlation was observed between hip and/or knee OA and lumbar spine BMD, but not femoral neck BMD*. SMAD3* was subsequently identified as one of the loci contributing to both traits. One could hypothesise that genetic correlation limited to lumbar spine BMD could reflect artefactual elevation of BMD in the presence of OA by spinal osteophytes. However, there has also been evidence for a genetic correlation between total body BMD measured in childhood and combined OA [30]; since total body BMD measurement during childhood is unaffected by OA, these findings suggest that there may be a true genetic correlation between BMD and OA, reflecting shared genetic aetiology. Shared genetic risk factors can indicate either that there are distinct effects of the variants on two separate traits or that one trait is causal for the other. Both scenarios would result in genetic correlation between the two traits, and further work is necessary to determine which is more likely.

### Wnt Signalling

Several genetic variants associated with BMD relate to the Wnt signalling pathway. *LRP5,* which as described above causes familial HBM [25, 26], is a Wnt co-receptor, whilst a common polymorphism at the *wnt16* locus is related to both BMD and fracture risk [35]. Wnt signalling also contributes to cartilage homeostasis and OA risk. For example, DOT1L, a histone methyltransferase, is involved in the downregulation of Wnt signalling as well as cartilage preservation [36]. Suggestive of a role of Wnt inhibition in reducing OA risk, a SNP in the *DOT1L* gene was associated with hip joint space width, an indirect measure of cartilage thickness, with genome-wide significance [37], and this same SNP has been associated with risk of hip OA [37, 38]. Further Wnt pathway inhibitors have been suggested to contribute to OA pathogenesis. For example, variants in the gene for the Wnt inhibitor, secreted frizzled-related protein 3, have been found to be associated with hip OA [39], and a novel small molecule Wnt inhibitor is being evaluated to treat knee OA [40].

The Wnt signalling pathway is complex, with different factors playing a predominant role in different tissue types. However, there is some evidence that certain factors within the Wnt pathway affect both the bone and cartilage. For example, mice with an *LRP5* deletion display increased cartilage degeneration compared to wild-type mice [41], although analyses by a different group reported the opposite [42]. In studies of the contribution of Wnt16 to OA aetiology, Nalesso et al. observed more severe OA in *Wnt16* knockout mice compared to that in wild type, suggesting Wnt16 has a role in preserving the cartilage after joint injury [43]. In contrast, Van den Bosch et al. found that *Wnt16* overexpression in the synovium of a mouse model led to increased expression of markers of cartilage degeneration [44], whereas Tornqvist et al. found no change in OA severity in transgenic mice overexpressing *Wnt16* in osteoblasts despite the increase in subchondral bone mass [45].

Sclerostin, a product of the *SOST* gene, was initially discovered as the underlying mutation in the HBM disorder sclerosteosis [46] and has since been targeted to treat postmenopausal osteoporosis in the form of romosozumab [47]. Sclerostin is produced by osteocytes and has been found to exert an important suppressive effect on osteoblastic bone formation [48]. Sclerostin binds to LRP5/LRP6 receptors to inhibit Wnt signalling and osteoblast activity. Several studies have examined a possible role of sclerostin in cartilage and OA development; however, studies of the contribution of sclerostin to OA have yielded somewhat conflicting results. *Sost* knockout mice have been found to show more severe cartilage lesions compared to wild-type mice [49]. This was supported by an earlier work of Chan et al. who provided further evidence that *SOST* is chondroprotective [50]. However, others have found no difference in cartilage lesions in *SOST* knockout mice [51], whilst most recently Zhou et al. identified more rapid OA development following surgical induction of OA in mice overexpressing *Sost* [52]. Sclerostin expression has been reported to be decreased in osteocytes of the subchondral bone in OA [50, 51, 53], and this decreased expression has been associated with subchondral bone thickening [50]. However, other studies have shown increased sclerostin staining in bone tissue and cartilage from tibial plateaus of patients who had undergone total knee replacement (TKR), compared to healthy control samples [54]. When investigating the relationship between circulating sclerostin and OA, one study found little evidence of an association between OA and serum sclerostin [55], whereas another reported reduced sclerostin levels in those with a higher radiographic severity of OA [56]. Consistent with a chondroprotective effect of sclerostin, in the HBM population described above, higher sclerostin levels were found to be associated with a lower risk of hip and thumb-base JSN [57].

Although these experimental studies provide some evidence that genes related to the Wnt pathway, such as *LRP5*, *WNT16* and *SOST*, are involved in the pathogenesis of OA, much of the evidence is conflicting. Moreover, it is often difficult to distinguish separate effects of a specific pathway on the cartilage, from those mediated by changes in the bone. A further question remains as to whether biological pathways shared between the bone and cartilage contribute to OA by altering bone formation or resorption. As discussed above, increased subchondral bone turnover and bone loss have been implicated in OA progression. Moreover, as well as regulating osteoblasts, the Wnt pathway and sclerostin are known to influence osteoclastic bone resorption [58, 59].

### Cathepsin K

Genetic variation in the osteoclast enzyme cathepsin K, which is required for bone resorption, was found to relate to the risk of developing OA in a recent GWAS, based on an association between a *CTSK* locus and prevalent OA [60]. This finding may reflect a protective effect of cathepsin K deficiency, and hence reduced bone resorption, on OA development, based on findings that a *Ctsk* knockout mouse showed delayed development of OA after surgery compared to wild-type mice [61]. Moreover, following a randomised controlled trial of a cathepsin K inhibitor, MIV-711, individuals with knee OA showed reduced MRI-assessed features of structural progression in the intervention groups, compared to placebo, as well as reduced levels of the cartilage breakdown marker uCTX-II [62]. However, rather than being mediated by altered bone resorption, these observations could reflect a direct influence of cathepsin K on cartilage: cathepsin K is also able to cleave type II collagen [63] and is expressed by human chondrocytes [63], and mRNA levels are increased in OA cartilage [64] and correlate with OA severity [65].

## Use of Genetic Studies to Examine the Causal Effect of BMD on OA Risk

The causal relationship between BMD and OA has also been examined using genetic variants related to BMD in the general population. For example, in a study of knee OA cases and controls, Yerges-Armstrong et al. identified an association between four BMD-associated SNPs (identified by Estrada et al. in the GEFOS cohort [66]) and knee OA [67]. These four SNPs associated with knee OA were among those most strongly associated with BMD, supporting a causal relationship (one would expect, in the case of a true causal effect, for the SNP-outcome association to increase as the SNP-exposure association increases). Additionally, one of the four SNPs was annotated to the *LRP5* gene implicated in familial HBM as described earlier [67]; the BMD increasing allele was associated with an increased odds of knee OA.

### Mendelian Randomisation (MR) Studies

MR is a more formal analysis framework for examining causal relationships [68]. Though MR can be limited by lack of power due to weak instruments, several genome-wide association study (GWAS) signals for BMD have been identified, making it feasible to explore causal relationships between BMD and OA. For example, the GEFOS consortium identified 56 loci associated with hip and/or lumbar spine BMD [66], and 518 loci were identified using BMD estimated from heel ultrasound (eBMD) in the UK Biobank [35]. Though the latter represents an indirect estimate of BMD, relatively high genetic correlation exists between the two traits, suggesting eBMD represents a reasonable proxy measure for BMD [69]. MR relies on several assumptions, including the absence of pleiotropy (i.e. a given genetic instrument relates to the outcome solely via a causal effect mediated by the exposure), which several methods have been developed to address [70]. As discussed above, genetic influences on BMD may be associated with OA risk via a separate chondrocyte pathway, or a shared ‘bone-forming’ tendency, both of which represent pleiotropy. Similarly, pleiotropy might exist between genetic factors related to BMD and other characteristics known to influence OA, such as BMI.

Two previous MR studies have examined causal relationships between BMD and OA, using common genetic variants associated with BMD. Evidence of a causal effect of BMD on OA was found using an instrument derived from a lumbar spine BMD GWAS [71]. In a recent MR study, Funck-Brentano et al. identified a causal effect of BMD on both hip and knee OA, using an instrument based on genetic association with femoral neck BMD [72]. To exclude pleiotropy involving BMI pathways, Funck-Brentano et al. performed sensitivity analyses following the removal of BMI-associated SNPs, after which equivalent results were obtained. A number of other sensitivity analyses were also performed to explore possible pleiotropy, including MR-Egger regression and weighted median analysis. However, when two traits such as BMD and OA are highly co-regulated, it is difficult to detect pleiotropy using these methods. For example, the weighted median method assumes that the majority of SNPs are non-pleiotropic, which might not be the case for highly correlated variables such as BMD and OA.

**Fig. 2:**
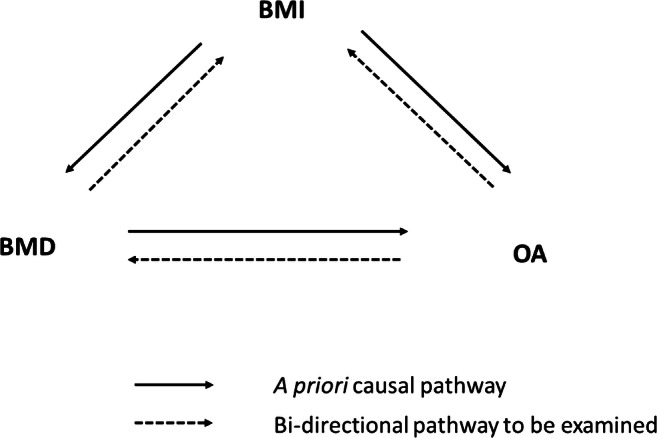
Hypothesised causal diagram for relationships between BMD, BMI and OA. In addition to the a priori causal pathway between BMD and OA suggested by Funck-Brentano [72], we propose a possible bi-directional causal pathway between OA and BMD. BMI is thought to act as a confounder via the biomechanical effects of excess weight on BMD and OA. In addition, bi-directional causal pathways might exist between BMD and BMI (through increased bone turnover) and between OA and BMI (through reduced physical activity)

As well as shared pathways between BMD and OA, causal pathways may also exist between these conditions and BMI. For example, given the suggested role for osteocalcin linking bone to energy metabolism [73], bone turnover may be related to adiposity, providing a further pathway between BMD and OA (Fig. 2). Hence, in a subsequent study, we examined each of these pathways in turn, accounting for possible relationships mediated by BMI using multivariable MR. Although we found evidence of an independent causal effect between BMD and OA, we also found evidence of a causal effect of OA on BMD, suggesting either a bi-directional relationship or pleiotropy that remained unaccounted for [74]. Multivariable effect estimates were similar to univariable estimates, providing little evidence of confounding by BMI. Therefore, though initial MR studies suggest that BMD has a causal effect on OA, the limited power of conventional pleiotropy tests, and our subsequent finding of bi-directional relationships, makes it difficult to exclude pleiotropy as an alternative explanation for these findings.

### Future Work

Looking to the future, several newer MR methods are emerging to detect and account for pleiotropy [75–78], the application of which should enable the causal relationship between BMD and OA to be examined more thoroughly. We have also started to develop methods to identify which pathways contribute to the shared genetics of these two traits. For example, if only a small number of pathway loci are shared between BMD and OA, this would imply that BMD is not causal for OA and that the branch point in the causal pathway that produces pleiotropic effects occurs relatively early on (equivalent to the scenario on Fig. 1b). Alternatively, if all pathways studied have proportionate effects on both BMD and OA, this suggests either scenario A or C in Fig. 1 that either BMD really is causal for OA or an intermediate phenotype proximal to BMD is the true causal risk factor for OA, consistent with the role of a ‘bone-forming’ phenotype underlying both raised BMD and osteophytic forms of OA in the HBM cohort (Fig. 1c). This area of study will be facilitated by the availability of large GWAS cohorts linked to information about OA sub-phenotypes including osteophytes, making it possible to establish whether similar relationships exist in the general population.

## Conclusions

As well as providing insights into shared genetic mechanisms between BMD and OA, bone genetics has been applied to examine the nature of relationships between BMD and OA. For example, the finding that individuals from our HBM cohort are at increased risk of developing OA raises the possibility that BMD plays a causal role in the development of OA. Although initial MR studies provided support for a causal effect of BMD on OA, further investigation has shown this to be more complicated, with either bi-directional or shared biological pathways also likely. Given the nature of the HBM phenotype and its genetic determinants, this may reflect the role of a ‘bone-forming’ phenotype in the development of OA. Further studies are required to dissect out the genetic pathways which contribute to the bone-OA relationship and to establish whether a similar ‘bone-forming’ phenotype underlies this relationship in more common forms of OA within the general population.

## References

[1] DALYs GBD, Collaborators H (2016). Global, regional, and national disability-adjusted life-years (DALYs) for 315 diseases and injuries and healthy life expectancy (HALE), 1990–2015: a systematic analysis for the Global Burden of Disease Study 2015. Lancet.

[2] Lories RJ, Luyten FP (2011). The bone-cartilage unit in osteoarthritis. Nat Rev Rheumatol.

[3] Antoniades L, MacGregor AJ, Matson M, Spector TD (2000). A cotwin control study of the relationship between hip osteoarthritis and bone mineral density. Arthritis Rheum.

[4] Burger H, van Daele PL, Odding E, Valkenburg HA, Hofman A, Grobbee DE (1996). Association of radiographically evident osteoarthritis with higher bone mineral density and increased bone loss with age. The Rotterdam Study. Arthritis Rheum.

[5] Chaganti RK, Parimi N, Lang T, Orwoll E, Stefanick ML, Nevitt M (2010). Bone mineral density and prevalent osteoarthritis of the hip in older men for the osteoporotic fractures in men (MrOS) study group. Osteoporos Int.

[6] Cooper C, Cook PL, Osmond C, Fisher L, Cawley MI (1991). Osteoarthritis of the hip and osteoporosis of the proximal femur. Ann Rheum Dis.

[7] Nevitt MC, Lane NE, Scott JC, Hochberg MC, Pressman AR, Genant HK, Cummings SR, STUDY OF OSTEOPOROTIC FRACTURES RESEARCH GROUP (1995). Radiographic osteoarthritis of the hip and bone mineral density. The Study of Osteoporotic Fractures Research Group. Arthritis Rheum.

[8] Hannan MT, Anderson JJ, Zhang Y, Levy D, Felson DT (1993). Bone mineral density and knee osteoarthritis in elderly men and women. The Framingham Study. Arthritis Rheum.

[9] Sowers M, Lachance L, Jamadar D, Hochberg MC, Hollis B, Crutchfield M, Jannausch ML (1999). The associations of bone mineral density and bone turnover markers with osteoarthritis of the hand and knee in pre- and perimenopausal women. Arthritis Rheum.

[10] Hart DJ, Cronin C, Daniels M, Worthy T, Doyle DV, Spector TD (2002). The relationship of bone density and fracture to incident and progressive radiographic osteoarthritis of the knee: the Chingford study. Arthritis Rheum.

[11] Zhang Y, Hannan MT, Chaisson CE, McAlindon TE, Evans SR, Aliabadi P, Levy D, Felson DT (2000). Bone mineral density and risk of incident and progressive radiographic knee osteoarthritis in women: the Framingham study. J Rheumatol.

[12] Bettica P, Cline G, Hart DJ, Meyer J, Spector TD (2002). Evidence for increased bone resorption in patients with progressive knee osteoarthritis: longitudinal results from the Chingford study. Arthritis Rheum.

[13] Spector TD, Conaghan PG, Buckland-Wright JC, Garnero P, Cline GA, Beary JF, Valent DJ, Meyer JM (2005). Effect of risedronate on joint structure and symptoms of knee osteoarthritis: results of the BRISK randomized, controlled trial [ISRCTN01928173]. Arthritis Res Ther.

[14] Burr DB, Gallant MA (2012). Bone remodelling in osteoarthritis. Nat Rev Rheumatol.

[15] Bruyere O, Dardenne C, Lejeune E, Zegels B, Pahaut A, Richy F, Seidel L, Ethgen O, Henrotin Y, Reginster JY (2003). Subchondral tibial bone mineral density predicts future joint space narrowing at the medial femoro-tibial compartment in patients with knee osteoarthritis. Bone.

[16] Gregson CL, Steel SA, O'Rourke KP, Allan K, Ayuk J, Bhalla A (2012). ‘Sink or swim’: an evaluation of the clinical characteristics of individuals with high bone mass. Osteoporos Int.

[17] Hardcastle SA, Gregson CL, Deere KC, Smith GD, Dieppe P, Tobias JH (2013). High bone mass is associated with an increased prevalence of joint replacement: a case-control study. Rheumatology (Oxford).

[18] Hardcastle SA, Dieppe P, Gregson CL, Arden NK, Spector TD, Hart DJ, Edwards MH, Dennison EM, Cooper C, Sayers A, Williams M, Davey Smith G, Tobias JH (2015). Individuals with high bone mass have an increased prevalence of radiographic knee osteoarthritis. Bone.

[19] Hardcastle SA, Dieppe P, Gregson CL, Hunter D, Thomas GE, Arden NK (2014). Prevalence of radiographic hip osteoarthritis is increased in high bone mass. Osteoarthr Cartil.

[20] Gregson CL, Paggiosi MA, Crabtree N, Steel SA, McCloskey E, Duncan EL, Fan B, Shepherd JA, Fraser WD, Smith GD, Tobias JH (2013). Analysis of body composition in individuals with high bone mass reveals a marked increase in fat mass in women but not men. J Clin Endocrinol Metab.

[21] Gregson CL, Hardcastle SA, Murphy A, Faber B, Fraser WD, Williams M, Davey Smith G, Tobias JH (2017). High bone mass is associated with bone-forming features of osteoarthritis in non-weight bearing joints independent of body mass index. Bone.

[22] Hartley A, Hardcastle SA, Paternoster L, McCloskey E, Poole KE, Javaid MK (2020). Individuals with high bone mass have increased progression of radiographic and clinical features of knee osteoarthritis. Osteoarthr Cartil.

[23] Radin EL, Paul IL, Rose RM (1972). Role of mechanical factors in pathogenesis of primary osteoarthritis. Lancet.

[24] Hardcastle SA, Dieppe P, Gregson CL, Arden NK, Spector TD, Hart DJ, Edwards MH, Dennison EM, Cooper C, Williams M, Davey Smith G, Tobias JH (2014). Osteophytes, enthesophytes, and high bone mass a bone-forming triad with potential relevance in osteoarthritis. Arthritis Rheumatol.

[25] Boyden LM, Mao J, Belsky J, Mitzner L, Farhi A, Mitnick MA, Wu D, Insogna K, Lifton RP (2002). High bone density due to a mutation in LDL-receptor-related protein 5. N Engl J Med.

[26] Little RD, Carulli JP, Del Mastro RG, Dupuis J, Osborne M, Folz C (2002). A mutation in the LDL receptor-related protein 5 gene results in the autosomal dominant high-bone-mass trait. Am J Hum Genet.

[27] Gregson CL, Bergen DJM, Leo P, Sessions RB, Wheeler L, Hartley A, Youlten S, Croucher PI, McInerney-Leo AM, Fraser W, Tang JCY, Anderson L, Marshall M, Sergot L, Paternoster L, Davey Smith G, Brown MA, Hammond C, Kemp JP, Tobias JH, Duncan EL, The AOGC Consortium (2020). A rare mutation in SMAD9 associated with high bone mass identifies the SMAD-dependent BMP signaling pathway as a potential anabolic target for osteoporosis. J Bone Miner Res.

[28] Gregson CL, Newell F, Leo PJ, Clark GR, Paternoster L, Marshall M, Forgetta V, Morris JA, Ge B, Bao X, Duncan Bassett JH, Williams GR, Youlten SE, Croucher PI, Davey Smith G, Evans DM, Kemp JP, Brown MA, Tobias JH, Duncan EL (2018). Genome-wide association study of extreme high bone mass: contribution of common genetic variation to extreme BMD phenotypes and potential novel BMD-associated genes. Bone.

[29] Gregson CL, Sayers A, Lazar V, Steel S, Dennison EM, Cooper C, Smith GD, Rittweger J, Tobias JH (2013). The high bone mass phenotype is characterised by a combined cortical and trabecular bone phenotype: findings from a pQCT case-control study. Bone.

[30] Hackinger S, Trajanoska K, Styrkarsdottir U, Zengini E, Steinberg J, Ritchie GRS, Hatzikotoulas K, Gilly A, Evangelou E, Kemp JP, Evans D, Ingvarsson T, Jonsson H, Thorsteinsdottir U, Stefansson K, McCaskie AW, Brooks RA, Wilkinson JM, Rivadeneira F, Zeggini E, arcOGEN Consortium, GEFOS Consortium (2017). Evaluation of shared genetic aetiology between osteoarthritis and bone mineral density identifies SMAD3 as a novel osteoarthritis risk locus. Hum Mol Genet.

[31] Valdes AM, Hart DJ, Jones KA, Surdulescu G, Swarbrick P, Doyle DV, Schafer AJ, Spector TD (2004). Association study of candidate genes for the prevalence and progression of knee osteoarthritis. Arthritis Rheum.

[32] Waarsing JH, Kloppenburg M, Slagboom PE, Kroon HM, Houwing-Duistermaat JJ, Weinans H, Meulenbelt I (2011). Osteoarthritis susceptibility genes influence the association between hip morphology and osteoarthritis. Arthritis Rheum.

[33] Yamada Y, Okuizumi H, Miyauchi A, Takagi Y, Ikeda K, Harada A (2000). Association of transforming growth factor beta1 genotype with spinal osteophytosis in Japanese women. Arthritis Rheum.

[34] Zhai G, Doré J, Rahman P (2015). TGF-β signal transduction pathways and osteoarthritis. Rheumatol Int.

[35] Morris JA, Kemp JP, Youlten SE, Laurent L, Logan JG, Chai RC (2019). An atlas of genetic influences on osteoporosis in humans and mice. Nat Genet.

[36] Monteagudo S, Cornelis FMF, Aznar-Lopez C, Yibmantasiri P, Guns LA, Carmeliet P, Cailotto F, Lories RJ (2017). DOT1L safeguards cartilage homeostasis and protects against osteoarthritis. Nat Commun.

[37] Castano Betancourt MC, Cailotto F, Kerkhof HJ, Cornelis FM, Doherty SA, Hart DJ (2012). Genome-wide association and functional studies identify the DOT1L gene to be involved in cartilage thickness and hip osteoarthritis. Proc Natl Acad Sci U S A.

[38] Evangelou E, Valdes AM, Castano-Betancourt MC, Doherty M, Doherty S, Esko T, Ingvarsson T, Ioannidis JPA, Kloppenburg M, Metspalu A, Ntzani EE, Panoutsopoulou K, Slagboom PE, Southam L, Spector TD, Styrkarsdottir U, Stefanson K, Uitterlinden AG, Wheeler M, Zeggini E, Meulenbelt I, van Meurs JB, arcOGEN consortium, the TREAT-OA consortium (2013). The DOT1L rs12982744 polymorphism is associated with osteoarthritis of the hip with genome-wide statistical significance in males. Ann Rheum Dis.

[39] Loughlin J, Dowling B, Chapman K, Marcelline L, Mustafa Z, Southam L, Ferreira A, Ciesielski C, Carson DA, Corr M (2004). Functional variants within the secreted frizzled-related protein 3 gene are associated with hip osteoarthritis in females. Proc Natl Acad Sci U S A.

[40] Deshmukh V, Hu H, Barroga C, Bossard C, Kc S, Dellamary L (2018). A small-molecule inhibitor of the Wnt pathway (SM04690) as a potential disease modifying agent for the treatment of osteoarthritis of the knee. Osteoarthr Cartil.

[41] Lodewyckx L, Luyten FP, Lories RJ (2012). Genetic deletion of low-density lipoprotein receptor-related protein 5 increases cartilage degradation in instability-induced osteoarthritis. Rheumatology (Oxford).

[42] Shin Y, Huh YH, Kim K, Kim S, Park KH, Koh JT, Chun JS, Ryu JH (2014). Low-density lipoprotein receptor-related protein 5 governs Wnt-mediated osteoarthritic cartilage destruction. Arthritis Res Ther.

[43] Nalesso G, Thomas BL, Sherwood JC, Yu J, Addimanda O, Eldridge SE, Thorup AS, Dale L, Schett G, Zwerina J, Eltawil N, Pitzalis C, Dell'Accio F (2017). WNT16 antagonises excessive canonical WNT activation and protects cartilage in osteoarthritis. Ann Rheum Dis.

[44] van den Bosch MH, Blom AB, van de Loo FA, Koenders MI, Lafeber FP, van den Berg WB, van der Kraan PM, van Lent PL (2017). Brief report: induction of matrix metalloproteinase expression by synovial Wnt signaling and association with disease progression in early symptomatic osteoarthritis. Arthritis Rheumatol.

[45] Tornqvist AE, Grahnemo L, Nilsson KH, Funck-Brentano T, Ohlsson C, Moverare-Skrtic S (2020). Wnt16 overexpression in osteoblasts increases the subchondral bone mass but has no impact on osteoarthritis in young adult female mice. Calcif Tissue Int.

[46] Brunkow ME, Gardner JC, Van Ness J, Paeper BW, Kovacevich BR, Proll S (2001). Bone dysplasia sclerosteosis results from loss of the SOST gene product, a novel cystine knot-containing protein. Am J Hum Genet.

[47] McClung MR, Grauer A, Boonen S, Bolognese MA, Brown JP, Diez-Perez A (2014). Romosozumab in postmenopausal women with low bone mineral density. N Engl J Med.

[48] Galea GL, Lanyon LE, Price JS (2017). Sclerostin’s role in bone’s adaptive response to mechanical loading. Bone.

[49] Bouaziz W, Funck-Brentano T, Lin H, Marty C, Ea HK, Hay E, Cohen-Solal M (2015). Loss of sclerostin promotes osteoarthritis in mice via beta-catenin-dependent and -independent Wnt pathways. Arthritis Res Ther.

[50] Chan BY, Fuller ES, Russell AK, Smith SM, Smith MM, Jackson MT, Cake MA, Read RA, Bateman JF, Sambrook PN, Little CB (2011). Increased chondrocyte sclerostin may protect against cartilage degradation in osteoarthritis. Osteoarthr Cartil.

[51] Roudier M, Li X, Niu QT, Pacheco E, Pretorius JK, Graham K, Yoon BRP, Gong J, Warmington K, Ke HZ, Black RA, Hulme J, Babij P (2013). Sclerostin is expressed in articular cartilage but loss or inhibition does not affect cartilage remodeling during aging or following mechanical injury. Arthritis Rheum.

[52] Zhou S, Ge Y, Li Y, Bao Z, Yao C, Teng H, Jiang Q (2017). Accelerated development of instability-induced osteoarthritis in transgenic mice overexpressing SOST. Int J Clin Exp Pathol.

[53] Wu L, Guo H, Sun K, Zhao X, Ma T, Jin Q (2016). Sclerostin expression in the subchondral bone of patients with knee osteoarthritis. Int J Mol Med.

[54] Abed E, Couchourel D, Delalandre A, Duval N, Pelletier JP, Martel-Pelletier J, Lajeunesse D (2014). Low sirtuin 1 levels in human osteoarthritis subchondral osteoblasts lead to abnormal sclerostin expression which decreases Wnt/beta-catenin activity. Bone.

[55] Theologis T, Efstathopoulos N, Nikolaou V, Charikopoulos I, Papapavlos I, Kokkoris P, Papatheodorou A, Nasiri-Ansari N, Kassi E (2017). Association between serum and synovial fluid Dickkopf-1 levels with radiographic severity in primary knee osteoarthritis patients. Clin Rheumatol.

[56] Mabey T, Honsawek S, Tanavalee A, Wilairatana V, Yuktanandana P, Saetan N, Zhan D (2014). Plasma and synovial fluid sclerostin are inversely associated with radiographic severity of knee osteoarthritis. Clin Biochem.

[57] Hartley A, Paternoster L, Murphy A, Hardcastle S, Tobias JH, Gregson CL (2018). Circulating sclerostin is associated with preserved joint space in non-weight bearing joints in a population enriched for high bone mineral density. J Bone Miner Res.

[58] Movérare-Skrtic S, Henning P, Liu X, Nagano K, Saito H, Börjesson AE, Sjögren K, Windahl SH, Farman H, Kindlund B, Engdahl C, Koskela A, Zhang FP, Eriksson EE, Zaman F, Hammarstedt A, Isaksson H, Bally M, Kassem A, Lindholm C, Sandberg O, Aspenberg P, Sävendahl L, Feng JQ, Tuckermann J, Tuukkanen J, Poutanen M, Baron R, Lerner UH, Gori F, Ohlsson C (2014). Osteoblast-derived WNT16 represses osteoclastogenesis and prevents cortical bone fragility fractures. Nat Med.

[59] Delgado-Calle J, Sato AY, Bellido T (2017). Role and mechanism of action of sclerostin in bone. Bone.

[60] Tachmazidou I, Hatzikotoulas K, Southam L, Esparza-Gordillo J, Haberland V, Zheng J (2019). Identification of new therapeutic targets for osteoarthritis through genome-wide analyses of UK Biobank data. Nat Genet.

[61] Kozawa E, Nishida Y, Cheng XW, Urakawa H, Arai E, Futamura N, Shi GP, Kuzuya M, Hu L, Sasaki T, Ishiguro N (2012). Osteoarthritic change is delayed in a Ctsk-knockout mouse model of osteoarthritis. Arthritis Rheum.

[62] Conaghan PG, Bowes MA, Kingsbury SR, Brett A, Guillard G, Rizoska B, Sjögren N, Graham P, Jansson Å, Wadell C, Bethell R, Öhd J (2020). Disease-modifying effects of a novel cathepsin K inhibitor in osteoarthritis: a randomized controlled trial. Ann Intern Med.

[63] Kafienah W, Brömme D, Buttle DJ, Croucher LJ, Hollander AP (1998). Human cathepsin K cleaves native type I and II collagens at the N-terminal end of the triple helix. Biochem J.

[64] Konttinen YT, Mandelin J, Li TF, Salo J, Lassus J, Liljeström M, Hukkanen M, Takagi M, Virtanen I, Santavirta S (2002). Acidic cysteine endoproteinase cathepsin K in the degeneration of the superficial articular hyaline cartilage in osteoarthritis. Arthritis Rheum.

[65] Kozawa E, Cheng XW, Urakawa H, Arai E, Yamada Y, Kitamura S, Sato K, Kuzuya M, Ishiguro N, Nishida Y (2016). Increased expression and activation of cathepsin K in human osteoarthritic cartilage and synovial tissues. J Orthop Res.

[66] Estrada K, Styrkarsdottir U, Evangelou E, Hsu YH, Duncan EL, Ntzani EE, Oei L, Albagha OME, Amin N, Kemp JP, Koller DL, Li G, Liu CT, Minster RL, Moayyeri A, Vandenput L, Willner D, Xiao SM, Yerges-Armstrong LM, Zheng HF, Alonso N, Eriksson J, Kammerer CM, Kaptoge SK, Leo PJ, Thorleifsson G, Wilson SG, Wilson JF, Aalto V, Alen M, Aragaki AK, Aspelund T, Center JR, Dailiana Z, Duggan DJ, Garcia M, Garcia-Giralt N, Giroux S, Hallmans G, Hocking LJ, Husted LB, Jameson KA, Khusainova R, Kim GS, Kooperberg C, Koromila T, Kruk M, Laaksonen M, Lacroix AZ, Lee SH, Leung PC, Lewis JR, Masi L, Mencej-Bedrac S, Nguyen TV, Nogues X, Patel MS, Prezelj J, Rose LM, Scollen S, Siggeirsdottir K, Smith AV, Svensson O, Trompet S, Trummer O, van Schoor NM, Woo J, Zhu K, Balcells S, Brandi ML, Buckley BM, Cheng S, Christiansen C, Cooper C, Dedoussis G, Ford I, Frost M, Goltzman D, González-Macías J, Kähönen M, Karlsson M, Khusnutdinova E, Koh JM, Kollia P, Langdahl BL, Leslie WD, Lips P, Ljunggren Ö, Lorenc RS, Marc J, Mellström D, Obermayer-Pietsch B, Olmos JM, Pettersson-Kymmer U, Reid DM, Riancho JA, Ridker PM, Rousseau F, lagboom PES, Tang NLS, Urreizti R, van Hul W, Viikari J, Zarrabeitia MT, Aulchenko YS, Castano-Betancourt M, Grundberg E, Herrera L, Ingvarsson T, Johannsdottir H, Kwan T, Li R, Luben R, Medina-Gómez C, Th Palsson S, Reppe S, Rotter JI, Sigurdsson G, van Meurs JBJ, Verlaan D, Williams FMK, Wood AR, Zhou Y, Gautvik KM, Pastinen T, Raychaudhuri S, Cauley JA, Chasman DI, Clark GR, Cummings SR, Danoy P, Dennison EM, Eastell R, Eisman JA, Gudnason V, Hofman A, Jackson RD, Jones G, Jukema JW, Khaw KT, Lehtimäki T, Liu Y, Lorentzon M, McCloskey E, Mitchell BD, Nandakumar K, Nicholson GC, Oostra BA, Peacock M, Pols HAP, Prince RL, Raitakari O, Reid IR, Robbins J, Sambrook PN, Sham PC, Shuldiner AR, Tylavsky FA, van Duijn CM, Wareham NJ, Cupples LA, Econs MJ, Evans DM, Harris TB, Kung AWC, Psaty BM, Reeve J, Spector TD, Streeten EA, Zillikens MC, Thorsteinsdottir U, Ohlsson C, Karasik D, Richards JB, Brown MA, Stefansson K, Uitterlinden AG, Ralston SH, Ioannidis JPA, Kiel DP, Rivadeneira F (2012). Genome-wide meta-analysis identifies 56 bone mineral density loci and reveals 14 loci associated with risk of fracture. Nat Genet.

[67] Yerges-Armstrong LM, Yau MS, Liu Y, Krishnan S, Renner JB, Eaton CB, Kwoh CK, Nevitt MC, Duggan DJ, Mitchell BD, Jordan JM, Hochberg MC, Jackson RD (2014). Association analysis of BMD-associated SNPs with knee osteoarthritis. J Bone Miner Res.

[68] Davey Smith G, Ebrahim S (2003). ‘Mendelian randomization’: can genetic epidemiology contribute to understanding environmental determinants of disease?. Int J Epidemiol.

[69] Kemp JP, Morris JA, Medina-Gomez C, Forgetta V, Warrington NM, Youlten SE, Zheng J, Gregson CL, Grundberg E, Trajanoska K, Logan JG, Pollard AS, Sparkes PC, Ghirardello EJ, Allen R, Leitch VD, Butterfield NC, Komla-Ebri D, Adoum AT, Curry KF, White JK, Kussy F, Greenlaw KM, Xu C, Harvey NC, Cooper C, Adams DJ, Greenwood CMT, Maurano MT, Kaptoge S, Rivadeneira F, Tobias JH, Croucher PI, Ackert-Bicknell CL, Bassett JHD, Williams GR, Richards JB, Evans DM (2017). Identification of 153 new loci associated with heel bone mineral density and functional involvement of GPC6 in osteoporosis. Nat Genet.

[70] Hemani G, Bowden J, Davey SG (2018). Evaluating the potential role of pleiotropy in Mendelian randomization studies. Hum Mol Genet.

[71] Zengini E, Hatzikotoulas K, Tachmazidou I, Steinberg J, Hartwig FP, Southam L, Hackinger S, Boer CG, Styrkarsdottir U, Gilly A, Suveges D, Killian B, Ingvarsson T, Jonsson H, Babis GC, McCaskie A, Uitterlinden AG, van Meurs JBJ, Thorsteinsdottir U, Stefansson K, Davey Smith G, Wilkinson JM, Zeggini E (2018). Genome-wide analyses using UK Biobank data provide insights into the genetic architecture of osteoarthritis. Nat Genet.

[72] Funck-Brentano T, Nethander M, Moverare-Skrtic S, Richette P, Ohlsson C (2019). Causal factors for knee, hip and hand osteoarthritis: a Mendelian randomization study in the UK Biobank. Arthritis Rheumatol.

[73] Lee NK, Sowa H, Hinoi E, Ferron M, Ahn JD, Confavreux C, Dacquin R, Mee PJ, McKee MD, Jung DY, Zhang Z, Kim JK, Mauvais-Jarvis F, Ducy P, Karsenty G (2007). Endocrine regulation of energy metabolism by the skeleton. Cell.

[74] Hartley A, Sanderson E, Granell R, Paternoster L, Zheng J, Southam L, Zeggini E, Gregson CL, Tobias JH (2020). Using multivariable Mendelian randomization to estimate the BMI-independent causal effect of bone mineral density on osteoarthritis. Osteoarthr Cartil.

[75] Cho Y, Haycock PC, Sanderson E, Gaunt TR, Zheng J, Morris AP, Davey Smith G, Hemani G (2020). Exploiting horizontal pleiotropy to search for causal pathways within a Mendelian randomization framework. Nat Commun.

[76] Morrison J, Knoblauch N, Marcus JH, Stephens M, He X (2020). Mendelian randomization accounting for correlated and uncorrelated pleiotropic effects using genome-wide summary statistics. Nat Genet.

[77] Slob EAW, Burgess S (2020). A comparison of robust Mendelian randomization methods using summary data. Genet Epidemiol.

[78] Verbanck M, Chen CY, Neale B, Do R (2018). Detection of widespread horizontal pleiotropy in causal relationships inferred from Mendelian randomization between complex traits and diseases. Nat Genet.

